# Strategies for recruiting Hispanic women into a prospective cohort study of modifiable risk factors for gestational diabetes mellitus

**DOI:** 10.1186/1471-2393-9-57

**Published:** 2009-12-11

**Authors:** Lisa Chasan-Taber, Renée T Fortner, Valerie Hastings, Glenn Markenson

**Affiliations:** 1 Division of Biostatistics & Epidemiology, Department of Public Health, School of Public Health & Health Sciences, University of Massachusetts, Amherst, MA, USA; 2 Division of Maternal-Fetal Medicine, Baystate Medical Center, Springfield, MA, USA

## Abstract

**Background:**

The purpose of this article was to describe effective strategies for recruitment of Hispanic women into a prospective cohort study of modifiable risk factors for gestational diabetes mellitus (GDM). Although Hispanic women have two to four times the risk of developing GDM compared with non-Hispanic white women, few GDM prevention studies have included Hispanic women.

**Methods:**

The study was conducted in the ambulatory obstetrical practices of Baystate Medical Center located in a socioeconomically and ethnically diverse city in Massachusetts. The study employed a range of strategies to recruit Hispanic women based on a review of the literature as well as prior experience with the study population.

**Results:**

Over a period of 32 months, a total of 851 Hispanic prenatal care patients were recruited. Among eligible women, 52.4% agreed to participate. Participants were young (70% <25 years), with low levels of education, and on public health insurance (81.5%); 88% were unmarried. Study design features such as use of bilingual recruiters, a flexible recruitment process, training recruiters to be culturally sensitive, use of culturally tailored materials, prescreening participants, participant compensation, seeking the cooperation of clinic staff, and continuous monitoring of recruitment goals emerged as important issues influencing recruitment.

**Conclusions:**

Findings suggest that investigators can successfully recruit pregnant women from ethnic minority groups of low socioeconomic status into observational studies. The study provides culturally appropriate recruitment strategies useful for practice-based settings recruiting Hispanic research participation.

## Background

Hispanic women are projected to have the highest birthrates for any minority group in the United States by the year 2009[1] and by 2030, are expected to be the largest minority group in the United States[2]. Despite the increase in population growth, substantial health disparities exist between Hispanic and non-Hispanic whites. Hispanic women have two to four times the risk of developing gestational diabetes mellitus (GDM) compared with non-Hispanic white women[3]. Important disparities also exist between the heterogeneous Latina subgroups. Hispanic women of Puerto Rican descent have an elevated risk of low birth weight and poor neonatal health outcomes as compared to other Hispanic groups [4-7]. Despite the increasing size of this population and the observed health disparities, relatively few health prevention studies on maternal and fetal disorders have included Hispanic women.

In 1994, the National Institutes of Health required recruitment plans for the inclusion of women and minorities in clinical research or scientific justification for their exclusion[8,9]. However, studies have found that conducting longitudinal studies involving Hispanics is challenging[10]. Hispanics in the United States are more likely than non-Hispanic whites to have low income and educational levels, factors which have been associated with nonparticipation in health research[11]. Lower levels of literacy and health literacy among Hispanics and a higher proportion of Spanish-only speaking households in a community have also been associated with reduced rates of participation[12]. In addition to such individual level factors, system-wide reasons cited for low participation among Hispanics include ineffective communication and informed consent procedures by research staff; lack of, or poor quality, incentives; cumbersome protocols; and failure to provide accessible sites for participation[13].

The majority of prior research has focused on traditional methods of community-based recruitment such as use of public databases, mass media advertising, or community health screening[13]. However, special challenges are encountered when recruitment targets a physician practice-based population. To our knowledge, no published studies have examined recruitment strategies for Hispanic pregnant women in a practice-based setting. Cost-effective culturally appropriate recruitment strategies that increase participation among Hispanic prenatal care patients are needed.

Proyecto Buena Salud is an ongoing prospective cohort study among pregnant women of Puerto Rican and Dominican descent with the major goal of evaluating modifiable determinants of GDM. This report describes the strategies that were developed to recruit Hispanic prenatal care patients from this practice-based setting, rates of recruitment over the first time, and characteristics of the study population. Recruitment into the study began in January 2006 and the results from the first 32 months are presented. This information should be valuable in planning studies and developing strategies to enhance recruitment in Hispanic pregnant populations.

## Methods

### Study Setting and Design

Proyecto Buena Salud is based in the ambulatory obstetrical practices of Baystate Health, an integrated health system in Western Massachusetts with a delivery rate of 4,500 infants annually. The study sites serve an ethnically and socioeconomically diverse population; approximately 57% of patients are Hispanic (predominantly from Puerto Rico), 17% are African-American, 23% are non-Hispanic white, and 3% are of other ethnicity. The overall recruitment goal was to enroll approximately 39 to 40 women per month, for a total of 1269 women over the 32 months of the study completed to date. We anticipated that rates of refusal would be 6%.

Prenatal care patients were recruited at their first prenatal care visit by bilingual recruiters. Reasons for exclusion included: diagnosis of type 2 diabetes, hypertension, heart disease, or chronic renal disease; treatment with medications thought to adversely influence glucose tolerance (i.e. prednisone or other corticosteroid); multiple gestation pregnancy; under age 16 or over 40 years of age; >20 weeks gestation. The population was limited to Caribbean Islanders (e.g., Puerto Ricans and Dominican Republicans) as they constituted the majority of the Latina population at the study site and have cultural practices and disease risks which differ substantively from other Latina groups (e.g., Mexican-Americans)[14]. The study protocol and written informed consent statements were approved by the Institutional Review Boards of the University of Massachusetts-Amherst and Baystate Medical Center.

At the time of enrollment, a baseline interview was administered using structured scales to assess physical activity patterns as well as psychosocial stress (i.e., anxiety, perceived stress, depression). Specifically, physical activity was measured by the Pregnancy Physical Activity Questionnaire (PPAQ),[15] perceived stress was measured using Cohen's Perceived Stress Scale (PSS-14),[16], trait anxiety was assessed using the Spielberger State-Trait Anxiety Inventory (STAI),[17] and depressive symptoms were assessed using the 10-item Edinburgh Postnatal Depression Scale (EPDS)[18]. All four scales have shown reasonable reliability and validity. Information on alcohol consumption, cigarette smoking, acculturation, and sociodemographic factors was also queried. Two subsequent interviews conducted in mid and late pregnancy updated this information and assessed dietary intake. After delivery, medical records were abstracted for medical and obstetric history, clinical characteristics of the current pregnancy, incident GDM, and birth outcomes.

### Recruitment Strategies

#### Bilingual Recruiters

Recruitment strategies were based on recommendations from the literature as well as our prior experience with the study population[19]. Our overall approach was to place dedicated, bilingual research staff in the clinical offices who focused on recruiting patients at the time of their regularly scheduled prenatal care visits. Physicians did not participate directly in recruitment activities and instead offered their support by agreeing to make their patients available for recruitment and by allowing their clinic staff to work with recruiters to identify potentially eligible participants.

#### Flexibility in Recruitment

The recruitment process was designed to be flexible to enhance participation. Due to potential interruption of the baseline interview (e.g., a woman is called into her medical exam) patients were asked to provide home, cell, and work telephone numbers and the name and telephone number of one relative or friend who did not live with them. If the baseline survey was not completed prior to the medical exam, the participant was asked if she could stay after her exam and a cafeteria voucher was provided to compensate her for her time. If women could not stay, the interview was completed via the telephone. Attempts were made to locate women not reached by telephone at their next prenatal care visit.

#### Recruiter Training

Recruiters were female and bilingual; the majority were native Spanish speakers. Recruiters were trained to give simple, clear information and be flexible and accommodating in recruiting women and performing interviews. Rapport was imperative for successful recruitment and was achieved through being respectful to the woman and culturally sensitive. Recruiters were trained to be credible by knowing the study objectives and design and to be responsive to reasons for reluctance to participate. Training involved role playing and shadowing of experienced recruiters. Recruitment strategies were discussed in monthly meetings with the Principal Investigator and on an ongoing basis with the project manager.

#### Culturally Tailored Materials

All study materials including eligibility screening forms, informed consent forms, HIPAA forms, questionnaires, posters, fliers, and patient handouts were translated into Spanish, back translated from Spanish to English, and again revised to create final Spanish versions. Translations utilized Spanish dialects spoken in the United States by individuals originating from Puerto Rico or the Dominican Republic.

#### Prescreening Processes

To increase efficiency, recruiters used a limited number of available demographic (i.e., date of birth) and medical characteristics (i.e., date of last menstrual period) provided on a daily roster of scheduled patients to generate a list of potential participants. Potential participants were defined as pregnant women who were, based on the daily roster, deemed to be between ages 16 and 40 years and early in pregnancy (<20 weeks gestation). The recruiters then targeted their efforts on locating potential participants at the time of their first prenatal care visit. Recruiters approached women in the waiting room and briefly explained the aims and procedures of the study. For those patients who were eligible and interested, the recruiters obtained written informed consent and proceeded to conduct the baseline interview.

#### Participant Compensation

A variety of items embossed with the study logo and contact information (e.g., small teddy bear, baby t-shirt, hat and bib) were given to participants at the time of recruitment to compensate women for their time. Women were also given a pedometer at enrollment. Gift certificates to a grocery store were provided for successful completion of the third interview and diet recall.

#### Cooperation from the Clinic Staff

Cooperation from the clinic staff was a critical recruitment strategy facilitated by designing study procedures to result in the least amount of interference with the daily functioning of the clinic. Before launching the study, the principal investigator, study obstetrician, and project manager held meetings with the clinic staff to explain the purpose of the study and receive feedback on study protocols. Periodically, throughout the duration of the study, the study obstetrician and project manager had breakfast meetings with the clinic staff to review the enrollment process. On a weekly basis, the Project Manager visited the clinics to address problems, demonstrate willingness to modify procedures to clinic needs, and show an appreciation for clinical staff involvement in the study.

#### Study Protocol

Women may be more likely to refuse participation in a study which requires a high participant burden[20]. Therefore, several adjustments were made to the protocol to reduce this burden. For example, questionnaires were interviewer-administered in Spanish or English based on patient preference to eliminate potential language or literacy barriers. In addition, the length of interview time was reduced by collecting medical and obstetric history from the patient's medical record as opposed to via self report at the time of the interview.

#### Monitoring of Recruitment Goals

A data management system was developed to track information about study recruitment. Reports generated by the system enabled recruiters to focus on the most likely eligible women. Reports included lists of: 1) patients currently enrolled to avoid approaching them for enrollment a second time, 2) patients ineligible for the study, 3) patients not interested in the study who did not want to be approached again, 4) patients with incomplete baseline interviews to be contacted by the telephone recruiters or in-person by recruiters at their next prenatal care visit, and 5) the total number of patients approached, screened, and reasons for ineligibility. These status reports were reviewed weekly such that potential problems could be identified and addressed in a timely fashion.

### Statistical Analysis

Recruitment rates were computed. Chi-square tests for categorical values were used to compare the sample to the greater Springfield/Massachusetts population. Data analysis was performed using SAS software, Version 9.1 (SAS Institute, Cary, NC).

## Results

### Response Proportions

Recruitment into the study began in January 2006 and the results from the first 32 months are presented. Using rosters of scheduled appointments, recruiters identified a total of 8,747 potential participants of whom 3,980 (45.5%) were not approached either because the patient did not attend her prenatal care appointment (55.0%), or because she was immediately called into her medical exam or the recruiter was with another participant (45.0%) (Table 1). A total of 3,142 (35.9%) of potential participants were ineligible either due to preexisting disease (4.0%), non-Puerto Rican/Dominican ethnic group (31.2%), or other exclusion criteria (i.e., <16 years, >20 weeks gestation, taking prednisone, or having twins/triplets) (64.8%). A total of 1,625 (18.6%) of potential participants were eligible. Among eligible women, 774 (47.6%) refused to participate and 851 (52.4%) agreed to participate and were successfully enrolled.

**Table 1:** Recruitment Rates; Proyecto Buena Salud, Western Massachusetts, 2006-2008.

	Total	
	n	%
Potential Participants	8747	100%
Not approached	3980	45.5%
No show/cancellation	*2189*	*55.0%*
Unable to contact*	*1791*	*45.0%*
Ineligible	3142	35.9%
Preexisting disease	*125*	*4.0%*
Not Puerto Rican/Dominican	*982*	*31.2%*
Other Ineligibility^†^	*2035*	*64.8%*
Eligible	1625	18.6%
Refused	*774*	*47.6%*
Recruited	*851*	*52.4%*

*Combination of missed interviewer busy and missed other reason.

† Younger than 16 yrs, >20 wks gestation, taking prednisone, twins/triplets.

### Sample Distribution

The participants in this study were young (approximately 70% less than age 25 years), with low levels of education (47.3% had not graduated high school) and on public health insurance (81.5%) (Table 2). Almost 88% of participants were unmarried. The majority of women preferred to speak English (79.3%) and slightly more than half of participants were born in the U.S. (52.5%). Approximately 40% of women reported alcohol consumption prior to pregnancy and 32% reported smoking prior to pregnancy, with 14% continuing smoking in pregnancy.

**Table 2:** Characteristics of Baseline Participants as Compared to the Greater Springfield/Massachusetts Area; Proyecto Buena Salud, Western Massachusetts, 2006-2008.

	Total Sample		Greater Springfield/Massachusetts Area		P-value
			
	N^a^	%	N	%	
Total sample	851	100.0			
Age (years)^b^					
16-24	586	69.7	356	61.9	0.02
25-29	156	18.6	139	24.2	
30-34	64	7.6	48	8.3	
35-40^c^	35	4.2	32	5.6	
Educational status^b^					
<High school	366	47.3	274	45.8	0.83
High school graduate	255	33.0	199	33.3	
Some college/graduate	153	19.8	125	20.9	
Health insurance^b^					
Private insurance	12	1.6	68	11.4	< 0.01
Public insurance	629	81.5	501	83.6	
Other	93	12.1	19	3.2	
Public and private insurance	4	0.5	0	0.0	
No insurance	30	3.9	6	1.0	
Don't know/refused	4	0.5	5	0.8	
Marital status^b^					
Single/divorced/separated/widowed	664	87.9	488	81.5	< 0.01
Married	91	12.1	111	18.5	
Language preference for speaking/reading^b^					
English	675	79.3	461	77.1	0.31
Spanish	176	20.7	137	22.9	
Birthplace^b^					
Puerto Rico/Dominican Republic^d^	334	47.8	215	35.9	0.01
United States	365	52.2	320	53.4	
Other			64	10.7	
Pre-pregnancy alcohol consumption^e^					
No	447	60.2	n/a	71.2	0.10
Yes	295	39.8	n/a	28.8	
Pre-pregnancy cigarette smoking^f^					
No	525	68.3	n/a	72.6	0.51
Yes	244	31.7	n/a	27.4	
Early pregnancy cigarette smoking^b^					
No	614	85.9	526	87.8	0.30
Yes	101	14.1	73	12.2	

^a^Numbers may not total to 851 due to missing data.

^b^Massachusetts Department of Public Health, Bureau of Health Statistics, Research and Evaluation, Registry of Vital Records and Statistics. N = 599 pregnant Hispanic women in Springfield, MA, 2006.

^c^Massachusetts Department of Public Health category age 35-39.

^d^Massachusetts Department of Public Health response options: Puerto Rico/US Territory, US, Other.

^e^Massachusetts Department of Public Health, Bureau of Health Statistics, Research, and Evaluation, Behavioral Risk Factor Surveillance Survey, N = 829 Hispanic females in MA, 2006.

^f^Massachusetts Department of Public Health, Bureau of Health Statistics, Research, and Evaluation, Behavioral Risk Factor Surveillance Survey, N = 843 Hispanic females in MA, 2006.

### Sample Representativeness

Table 2 compares the cohort to women from the greater Springfield/Massachusetts area[21]. Women enrolled in Proyecto Buena Salud did not differ from Springfield area pregnant women in terms of education, language preference, or early pregnancy cigarette smoking. However, participants were slightly more likely to be younger, less likely to have private insurance, more likely to be unmarried, and more likely to be born in Puerto Rico or the Dominican Republic. As compared to female Hispanic Massachusetts residents in the Behavioral Risk Factor Surveillance Survey,[22] participants in Proyecto Buena Salud did not differ significantly in terms of pre-pregnancy alcohol consumption and cigarette smoking.

### Rates Over Time

**Figure 1:**
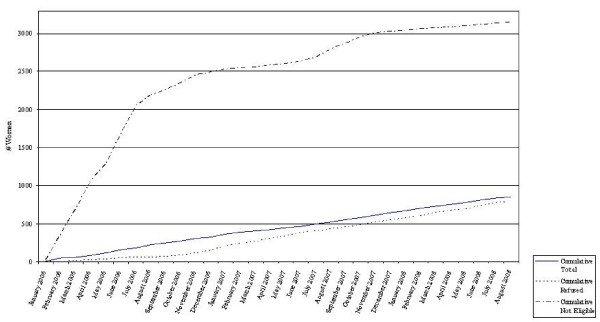
**Enrollment into Proyecto Buena Salud, Western Massachusetts, 2006-2009**.

Recruitment rates were fairly constant over the study period (Figure 1) with an average recruitment rate of 26.8 ± 7.5 women per month. Similarly, refusal rates remained fairly constant over the study period although a slight increase over the course of the study can be observed. Ineligibility rates grew rapidly at the onset of the study and then achieved a steadier slope after 1 year of recruitment. This was likely due to a 'training effect' whereby the recruiters became more adept over time at pre-screening potential participants. That is, they became more skilled at scanning the rosters and differentiating potentially eligible from ineligible subjects based on gestational age, birth date, and type of visit.

## Discussion

Strategies used to recruit pregnant Hispanic women of varying educational and socioeconomic status into a longitudinal cohort study, Proyecto Buena Salud, were described. Findings add to the sparse collection of strategies to include Hispanic women in research on areas of maternal/fetal health disparities. Several techniques were implemented to promote recruitment from this clinical setting. These included 1) use of bilingual recruiters, 2) a flexible recruitment process, 3) training recruiters to be culturally sensitive, 4) use of culturally tailored materials, 5) prescreening of participants, 6) compensating participants, 7) seeking cooperation from the clinic staff, and 8) continuously monitoring recruitment goals. Like other investigators,[13,23] we utilized multiple different but complementary strategies. In addition, pregnancy is a finite time interval during which women may be more motivated to learn about the impact of their behaviors on their own health as well as that of their future offspring[23].

The overall recruitment goal of Proyecto Buena Salud was to enroll approximately 39 to 40 women per month, for a total of 1269 pregnant Hispanic women over the first 32 months of the study. Our observed recruitment rates were lower at 27 women per month, for a total of 851 women over the first 32 months of the study. The inability to meet projected recruitment goals was due, in part, to higher than anticipated refusal rates which were 48% of eligible women as opposed to a projected refusal rate of 6%. Prior data on participation rates in pregnancy cohorts is sparse. However, Savitz et al. reported recruiting 57% of eligible pregnant women into the Pregnancy, Infection, and Nutrition (PIN) Study[24]. We observed slightly lower, but comparable, recruitment rates of 52.4% of eligible women. Although similar in design to Proyecto Buena Salud, the PIN study was conducted among predominantly non-Hispanic white and African-American women and also required a blood sample.

A recruitment challenge unique to clinical settings is cancelled appointments. Among the potential participants that were not approached in the clinic, 55.0% did not show for their scheduled prenatal care appointment or had rescheduled. The fact that these appointments were not study-related suggests that the traditional approach of asking potential participants to make separate (non medical) trips to the clinic would not be feasible for this population. Our study population was predominantly young and unmarried. This is consistent with prior studies which suggest that approximately 40% of Hispanic young girls, especially in low income families, drop out of school by eighth grade, are frequently in partnered relationships, and begin childbearing early[25]. Low attendance to prenatal visits may therefore be due to personal and child sickness, domestic tasks, unanticipated employment opportunities, and partner restrictions.

It is also possible that the projected recruitment goals of Proyecto Buena Salud were overly ambitious. A recent review of participation rates in epidemiologic surveys,[20] found that participation rates have been declining during the past 30 years with even steeper declines in recent years. For example, national epidemiologic surveys conducted among the general population such as the 2005 Behavioral Risk Factor Surveillance Survey and Multi-Ethnic Study of Atherosclerosis, initiated in 2000, observed recruitment rates of 51.1% and 59.8%, respectively[20].

Our strategies to recruit Hispanic pregnant participants had several strengths. The clinical staff understanding of the study design and sense of involvement helped maintain the integrity of the research effort. The use of dedicated research staff from similar cultural backgrounds who were trained to be culturally sensitive established the legitimacy of the study and allayed potential fear about the use of information collected. Prior research has shown that the race, ethnicity, and sex of a recruiter can affect a respondent's level of cooperation[12]. The use of bilingual recruiters as well as Spanish versions of study materials helped to address linguistic isolation.

By focusing on patients from physician practices who had regularly scheduled prenatal care visits, research demands on participants' time, effort, and travel were minimized. In addition, by collecting selected data from the medical records after delivery, participant burden was reduced. The option to complete the enrollment process in one of multiple ways, by telephone or at a subsequent prenatal care visit, was also viewed as removing critical barriers to enrollment.

It has been suggested that monetary incentives may have a greater impact on the decisions of minority, low education, and low income individuals to participate in research studies. However, other research suggests that monetary incentives encourage participation among respondents with higher education and income who have a greater demand to be compensated for their time[20]. Our study provided women with a combination of gifts for the baby as well as retail gift certificates; recruiters indicated that this was a critical factor in women's decision to participate.

Our strategies to recruit participants had several limitations. This study found, as have others,[24] that ensuring that each eligible patient was approached by the recruiter was difficult given staffing constraints and the competing, higher priority need for the medical exam. Recruiters often missed potential participants because they were busy with other participants. This is due, in part, to the clustered scheduling of new prenatal care patients at certain times of the day. In contrast, increasing the number of recruiters led to overstaffing for the remainder of the day. Having regular clinic staff perform recruiting was considered, but ruled out, because the protocol was too complex. In addition, the logistical challenges that occur in a busy clinic pose challenges to clinic staff that are already overburdened. At times, over the course of recruitment, clinic staff considered the presence of recruiters an intrusion which may have also impacted recruitment rates in unmeasurable ways.

Women who were located in the clinic and approached for recruitment may differ in substantive ways from women who were not located. Due to HIPAA privacy regulations, information could not be collected on women who refused to participate or were not eligible or missed. However, comparisons between our sample and Hispanic women from the greater Springfield/Massachusetts area, the source population from which this cohort arose, were conducted. While mail surveys of women after delivery[26] have found that women who choose to participate in health research studies are healthier overall than those who choose not to participate, cohort studies of pregnant women have not found this to be true[24]. Similarly, this study found no substantial differences in the socio-demographics and risk profiles between enrolled women and women in the greater area indicating that the current sample is largely representative. However, it is important to note that findings based on the contacted population may not be generalizable to this larger population.

Lastly, a variety of recruitment strategies were used which likely had a synergistic impact on recruitment. Due to their implementation at overlapping times, it cannot be determined to what degree each recruitment technique would succeed individually. Due to the prospective cohort study design used in the current study, it was not possible to determine whether this intensive recruitment strategy would be more effective than a less intensive strategy. A randomized trial would be required to test the impact of these recruitment strategies on recruitment rates. In addition, qualitative research exploring why some strategies were effective, while other strategies were less effective, would also informative.

## Conclusions

In summary, findings from Proyecto Buena Salud document that investigators can recruit pregnant women from ethnic minority groups of low socioeconomic status into observational studies. The commitment of Hispanic women to improving their health and readiness to contribute to developing new knowledge is an invaluable lesson learned. Future studies examining the challenges and strategies presented in this paper could enhance minority participation in observational pregnancy cohorts.

## Competing interests

The authors declare that they have no competing interests.

## Authors' contributions

LCT conceived of the study, and participated in its design and coordination and drafted the manuscript. RF and GM participated in the design and coordination of the study. RF and VH performed the statistical analysis. All authors read and approved the final manuscript.

## Pre-publication history

The pre-publication history for this paper can be accessed here:

http://www.biomedcentral.com/1471-2393/9/57/prepub
